# Epicardial Atrial Fibrillation Ablation and Left Atrial Appendage Amputation in a Patient with Congenital Bicuspid Aortic Valve and Catheter-Induced Cardiac Tamponade

**DOI:** 10.21470/1678-9741-2019-0244

**Published:** 2020

**Authors:** Yaming Shi, Yongzhong Zong

**Affiliations:** 1 Department of Cardiology, Yancheng Third People’s Hospital, Yancheng Hospital Affiliated to Medical College of Southeast University, Jiangsu, China.

**Keywords:** Atrial Appendage, Atrial Fibrillation, Catheter Ablation, Thoracic Surgical Procedures, Punctures, Amputation, Drainage, Cerebrovascular Circulation

## Abstract

Early recognition and rapid and appropriate treatment of cardiac tamponade are mandatory to prevent the irreversible deterioration of cerebral perfusion and other important organs. In this study, cardiac tamponade was induced by inadvertent transseptal puncture, which was managed with pericardial drainage and surgical repair in a patient with symptomatic paroxysmal atrial fibrillation. Epicardial atrial fibrillation ablation and left atrial appendage amputation were also performed at the same time.

**Table:** 

Abbreviations, acronyms & symbols	
AF	= Atrial fibrillation
LAA	= Left atrial appendage

## INTRODUCTION

Atrial fibrillation (AF) is the most common type of arrhythmia. It can impair quality of life and easily lead to stroke and increase the risk of cardiac death. Antiarrhythmic drugs, radiofrequency ablation and surgical ablation are widely used in rhythm conversion of AF. However, radiofrequency catheter ablation for AF is one of the most complex interventional electrophysiological procedures, which invokes catheter manipulation and ablation in the delicate thin-walled atria, close to other vital organs and structures that may be affected through collateral damage by its nature^[1]^. In this study, cardiac tamponade was induced by inadvertent transseptal puncture, which was managed with pericardial drainage and surgical repair in a patient with symptomatic paroxysmal AF and congenital bicuspid aortic valve. Epicardial AF ablation and left atrial appendage amputation (LAA) were also performed at the same time. During the follow-up period of one year after discharge, the patient was asymptomatic and had no recurrence of AF.

### Case Presentation

**Fig. 1:**
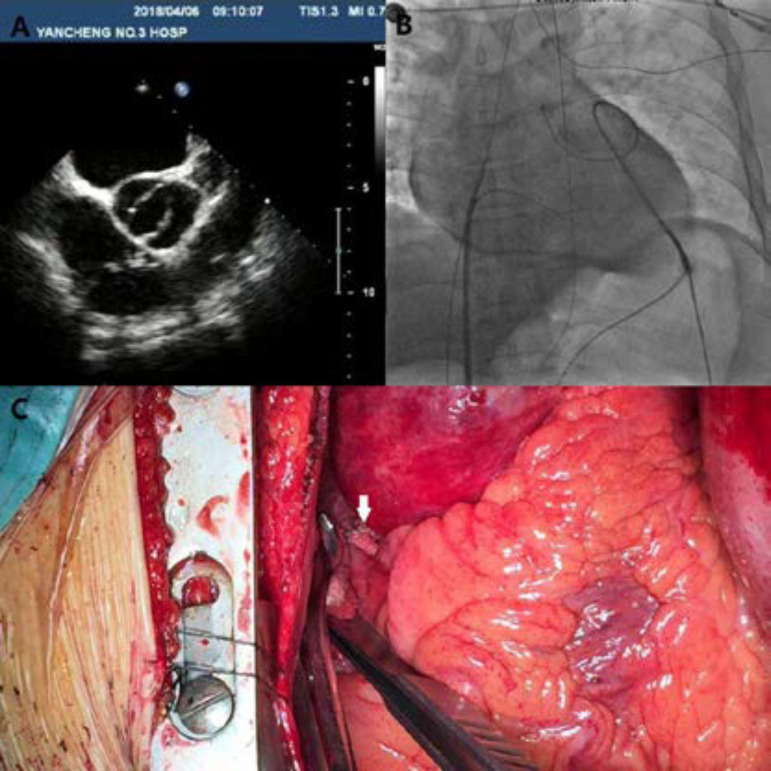
Images of transesophageal echocardiogram, chest X-ray and thoracic surgery. A: Transesophageal echocardiogram reavealed congenital bicuspid aortic valve. B: Guidewire was found to enter the thoracic aorta and pericardial drainage was performed via apical puncture with the Seldinger technique. C: Hematoma in the region of right atrium roof and the thoracic aorta were seen and the ongoing bleeding was managed by surgical repair (white arrow).

A 46-year-old male patient was admitted to our department for ablation of symptomatic paroxysmal AF. Physical examination upon admission revealed blood pressure of 126/84 mmHg, respiratory rate of 15/min, and heart rate of 72 bpm/min, clear lungs, irregular heart sounds and no murmur. On admission, chest X-ray was normal, and the electrocardiography showed AF. Transesophageal echocardiogram demonstrated congenital bicuspid aortic valve (Figure 1A) and transthoracic echocardiogram showed normal left ventricular systolic function with ejection fraction of 60%. During the catheter ablation process, catheters were routinely inserted into the right ventricle and coronary sinus. However, misdirected transseptal puncture was diagnosed when the guidewire was found in the thoracic aorta. About ten minutes later, chest fluoroscopy showed a small amount of pericardial effusion. Twenty minutes later, the bleeding increased and the patient’s condition became unstable; therefore, pericardial drainage of 600 ml of blood was performed via apical puncture with the Seldinger technique (Figure 1B). After one hour, bleeding was decreased, and the patient was immediately transferred to the operating room, maintaining the atrial septum puncture sheath at the puncture site.

### Description of Employed Technique

A hematoma was observed in the thoracic aorta region and the ongoing bleeding was managed by double purse suture with tapetum. Additionally, the ongoing bleeding in the region of right atrial roof was similarly managed by mattress suture with tapetum (Figure 1C, white arrow). Then, the left and right pulmonary veins were carefully separated and circular ablation of both right and left pulmonary veins was performed using a bipolar ablation clamp (OLL2 Isolator Synergy Clamp, AtriCure Inc., Mason, OH, USA). All ablations were doubled to ensure electrical blockage. In the meantime, the LAA was resected and oversewn to prevent thrombus formation. No further complications were observed during admission. He had recovered and was discharged without further sequelae after two weeks. The patient was followed at 1 month, 3 months, 6 months, 9 months and 12 months after surgery, was asymptomatic and had no recurrence of AF, which was confirmed by 24-hour ambulatory electrocardiogram.

### Comments

Patients with AF are at risk of stroke, tachycardia-induced cardiomyopathy and medical treatment-related complications. Therefore, increasing attention has been given to the development of interventional treatments for AF. Catheter ablation is preferred to surgical ablation in most cases; however, the latter is favored if catheter ablation is unlikely to succeed or not feasible. Notably, cardiac tamponade remains the potentially life-threatening complication associated with AF ablation^[2]^. An early sign of cardiac tamponade is a reduction in the excursion of the cardiac silhouette on fluoroscopy, along with a simultaneous decline in systemic blood pressure. Early recognition and rapid and appropriate treatment of cardiac tamponade are usually mandatory to prevent the irreversible deterioration in perfusion of the brain and other important organs. Percutaneous drainage can be performed either under fluoroscopic guidance based on anatomic landmarks or under echo guidance^[3]^. After initial aspiration, blood pressure can promptly return to normal. In rare cases, percutaneous drainage may be inadequate in the presence of a tear, and surgical drainage and repair would be necessary^[4]^. A recent meta-analysis reported that 16% of cardiac tamponade cases required surgical intervention^[5]^. For this reason, AF ablation procedures should only be performed in hospitals equipped or prepared to manage these types of emergencies, with access to emergency surgical support when required. In this case, it is also important to maintain the sheath at the puncture site until surgical repair can be performed, since the sheath can decrease bleeding.

Pulmonary veins can be surgically isolated by the endocardial route after opening the left atrium or by the epicardial route. Cox maze **III** and **IV** are the most successful therapeutic options from the surgical standpoint^[6]^, but the technical complexity and degree of invasiveness of such approaches have restricted their wide acceptance among patients and cardiologists. Therefore, there has been an increasing interest in the less invasive off-pump surgical techniques for AF treatment in the last decade. Minimally invasive and epicardial-endocardial ablation procedures have been developed, which can be ascribed to the demand for open heart surgery and the morbidity associated with the Cox maze surgical procedure^[1]^. The epicardial method is mainly advantageous because the pulmonary veins can be isolated without cardiopulmonary bypass, thus simplifying the surgical procedure and reducing the risk of complications. Benussi et al.^[7]^ had described an original radiofrequency ablation technique to treat AF in patients undergoing mitral valve surgery, in which two encircling lesions around the right and left pulmonary vein ostia were performed epicardially, and the endocardial lesions connecting the encircling between them and to the mitral valve annulus were also completed. At a mean follow-up of 11.6±4.7 months, 76.9% patients were in a stable sinus rhythm^[7]^. Accordingly, epicardial AF ablation was performed after surgical repair in this case.

Retrospective evaluation has suggested that LAA is responsible for up to 90% of strokes in patients with AF and nonrheumatic heart disease^[8]^. LAA has an anatomic structure that is quite favorable for thrombus formation in AF patients who often experience limited contraction and stagnant blood flow. Surgical treatment of LAA has been increasingly performed over the past few years. According to ACC/AHA/ESC guidelines, LAA should be excluded when mitral valve or other cardiac surgery is performed in AF patients. Amputated LAA was performed after the patient had undergone epicardial AF ablation in our case.

**Table:** 

Authors' roles & responsibilities	
YS	Substantial contributions to the conception or design of the work; or the acquisition, analysis, or interpretation of data for the work; drafting the work or revising it critically for important intellectual content; final approval of the version to be published
YZ	Substantial contributions to the conception or design of the work; or the acquisition, analysis, or interpretation of data for the work; drafting the work or revising it critically for important intellectual content; final approval of the version to be published
